# Changing the Narrative for Nursing Globally

**DOI:** 10.5334/aogh.2807

**Published:** 2020-04-06

**Authors:** Nancy Bolan, Yolanda Ogbolu

**Affiliations:** 1 Office of Global Health, University of Maryland School of Nursing, Baltimore, MD, US; 2 Partnerships, Professional Education and Practice, University of Maryland School of Nursing, Baltimore, MD, US

## Abstract

Worldwide celebration of the Year of the Nurse and the Midwife boosts innovative efforts by academic nursing schools to change the narrative for the nursing profession globally. These innovations have the potential to disrupt narratives that perpetuate negative perspectives and to replace them with counter-narratives that elevate, motivate and empower the professions to the benefit of health service delivery and health systems globally.

## Background

Motivated and empowered health professionals are critical to promoting health equity and saving lives globally [1]. Evidence shows that health worker numbers and quality are positively associated with population health outcomes [2]. Since nurses and midwives comprise over half of the health workforce in many countries, their work is clearly critical in delivering essential health services, strengthening the health system, and assuring the attainment of global health goals such as universal health coverage and the Sustainable Development Goals [3,4]. However, nurses are undervalued in many parts of the world, and their work has been characterized as “invisible” [5]. Bowker, Star and Spasser argue that “only work that is visible can truly be identified as valuable” [6]. We suggest that nurses can change archaic narratives about the profession. In doing so, they are positioned to influence public opinion and positively impact global health.

Narratives are described as a system of related stories that are articulated and refined over time to represent a central idea or belief; they reflect a shared interpretation of how the world works [7]. The Narrative Initiative writes, “Who holds power and how they use it is both embedded in and supported by dominant narratives; successful narrative change shifts power, as well as dominant narratives” [8]. Power derived from narratives has influence over those who set the norms that shape society and makes the presence of an issue powerful in a way that forces change in the status quo. Narrative change requires gathering, sharing and uplifting new alternative stories and can be utilized to shape consciousness and catalyze transformational change [7]. We propose that narrative change is at work in 2020, declared by the World Health Organization as The Year of the Nurse and the Midwife [9]. This recognition lifts up the past, present and future of nursing, celebrating and elevating the nursing workforce globally, thus motivating and empowering nurses everywhere.

Nursing’s inception as a modern profession was linked to global health. Florence Nightingale and Louisa Parsons, Nightingale’s student, both leaders in global health, recognized the need to strengthen nursing when they founded nursing schools on two continents. Nightingale, the leader of global health nursing, led reductions in mortality and morbidity by establishing public health and epidemiology programs on the front during the Crimean War. Parsons, selected by Nightingale, accompanied British soldiers on two campaigns into Egypt and the Sudan between 1883 and 1885, and was awarded British nursing’s ultimate accolade for her service, the Royal Red Cross [10]. Nightingale founded a nursing school in London, while Parsons founded The University of Maryland School of Nursing (UMSON) in Baltimore, Maryland. Both groundbreaking nurse leaders changed the narrative of the nursing profession of their day [11].

Today, academic nursing continues to advance innovative initiatives that disrupt archaic narratives that serve to perpetuate negative perspectives and gender-related biases rendering nursing invisible and downplaying the global importance of the profession. Innovative global health initiatives present opportunities to advance counter-narratives that elevate, motivate and empower, resulting in the shifting of cultural consciousness towards an appreciation of the value of nurses, critical for improved global health equity and improved outcomes worldwide.

## Setting the Scene

Health care is a labor-intensive service industry, and health providers personify the health system’s core values – they heal and care for people, mitigate pain and suffering, prevent disease and reduce risk [3]. Capable, motivated and supported health workers are essential for overcoming bottlenecks to attain national and global health goals. Since service quality is directly mediated by workers’ willingness to apply themselves to their tasks, health sector performance is dependent on worker motivation [1]. As noted by the World Health Organization (WHO) in their 2016–2020 strategy for strengthening nursing and midwifery, these professionals can completely change how health services are provided if they are supported by health systems [2]. Workforce policies that support health professionals are thus essential elements needed for better functioning health systems such that they will be able to respond to current and emerging health problems [3].

However, social, economic and professional barriers have been identified as negatively impacting the professions, which are largely dominated by women [12,13]. A global policy guidance report concluded that gender inequality and lack of female empowerment was the most significant barrier to the advancement of the midwifery profession [14]. Similarly, WHO noted in their seminal 2006 World Health Report on human resources for health that at the time of publication, typically, more than 70% of doctors were male while more than 70% of nurses were female – a marked gender imbalance despite a changing gender landscape [3].

The “Lancet Commission” Report on the Education of Health Professionals for the 21st Century called for creating a transformative and interdependent professional education system for health professionals with the goal of producing “enlightened change agents” to improve population health equity within strengthened health systems. To achieve that goal, one essential action proposed in the document was strengthening global learning, which is an approach to learning that recognizes the importance of linking people’s lives throughout the world [15,16].

Through innovative initiatives focused on educating nurses and midwives as global health experts and advocates, together with other inter-dependent health professionals, academic nursing schools are building the capacity of nurses locally and globally to be enlightened change agents, empowered to change the narrative and lead the charge for strengthening health systems and saving lives worldwide. Global learning, interprofessional educational approaches, and partnership are key principles promulgated by the WHO for strengthening nursing and transforming health professional education that are exemplified by innovative approaches to educating global health nurses and other health professionals. These strategic efforts support global efforts to change the narrative and lift nursing and midwifery to their rightful place given their importance worldwide.

## Strategic and Innovative Initiatives

**1) Global learning** is closely allied with mutual learning, a process of learning between people and health services around the world [17]. The ideal of cooperation to improve global health has become increasingly important as the world becomes more interconnected and interdependent. Health systems in the USA and around the world are facing similar challenges and common goals to improve population health, including the need to 1) emphasize health promotion and disease prevention; 2) build models of multisectoral collaboration for health; 3) engage communities and patients in their health and healthcare, and 4) develop sustainable and affordable models of care [17]. Given the parallels of global health system challenges, solutions used to address global challenges may also be very similar. Various international programs at academic nursing schools have been strategically designed for both global and local impact and ownership, preparing students to face an interconnected world through a better understanding of different cultures. In Baltimore, we are implementing a global learning program from Brazil (Saude Crianca) to address social isolation for vulnerable families of young children. This program is modeling the ideals of “ownership” with collaborative leadership, local community organization engagement, and involvement of local and global beneficiaries in all aspects of the program implementation [18].

Global health nurses often say that they always learn more than they give to nurses in developing countries. Global learning offers an opportunity to acknowledge the innovation and creativity of nurses in low resource settings. Recognizing the substantial contributions of our nurse colleagues around the world, we elevate the status of nurses, making nurses visible, disrupting negative narratives, and bringing forth the best evidence to improve the health of local and global populations.

**2) Interprofessional education** is a key approach recommended for both the undergraduate and graduate educational levels by WHO resolutions on strengthening nursing and midwifery services that have been passed by the World Health Assembly (WHA64.7) [2,19,20]. Interprofessional education (IPE) is defined by WHO as the process by which students from various professional programs study together during certain parts of their education to augment collaboration and teamwork to drive mutual understanding of the others roles, core competencies, and language and thinking. Research reveals that IPE improves communication and team-working skills and increases health worker self-confidence and appreciation of the roles of other health colleagues [19].

The UMSON champions interprofessional education in the preparation of the next generation of global health professionals through their Global Health Certificate Program, in which interprofessional health faculties collaborate with students from all professional schools at the University of Maryland Baltimore campus (including students from the Schools of Nursing, Medicine, Pharmacy, Law and Social Work), as well as global health partners on the ground. The certificate program recognizes the need to combine didactic learning experiences with practical global health field experiences in resource-limited countries in the global south or resource-limited settings in the United States. An example of an interprofessional global health field experience was a partnership with the Institute of Human Virology-Nigeria that involved three successive years of evaluation of one of the first significant task shifting efforts in HIV/AIDS care in a large teaching hospital in Abuja, the capital of Nigeria. Task shifting is a strategy to increase the capacity of health care systems to provide care and treatment [21]. Given that task shifting involved a major shift in interprofessional relationships, an interprofessional approach was needed to evaluate the question comprehensively. Successful interprofessional education programs have the potential to shift traditional hierarchical power dynamics in healthcare, increase the visibility of nursing contributions, and advance a positive narrative related to the status of the nurse in interdisciplinary collaborations in education, research and clinical practice.

**3) Partnership** is another key guiding principle for strengthening nursing and midwifery, in alignment with the principles of the Global Strategy on Human Resources for Health: Workforce 2030 [2,22]. Academic nursing programs build meaningful international partnerships through various mechanisms such as international nurse exchange programs. These programs offer specialized training programs for diverse groups of international nurses such as international visiting scholar (IVS) programs, which welcome nursing research, education and practice scholars from around the world to different institutions in the United States. These programs strive to expand individuals’ global health nursing capacity as leaders, educators and research scholars, with a focus on building new partnerships and initiatives.

Nurse capacity building through IVS programs has a rich history, including Florence Nightingale’s appointment to take nurses from England to Turkey in 1853, and the creation of the International Council of Nurses in 1899 [23]. Continuing the legacy, US-based nursing programs have adeptly accepted the challenge to strengthen global nursing capacity through a variety of IVS programs. Visiting research scholars receive structured experiences and engage with expert faculty mentors to refine project proposals, analyze data, synthesize findings, and develop and submit collaborative manuscripts. Educational scholars seek opportunities to observe clinical and academic courses, attend presentations, trainings, and examine curriculum development processes with faculty mentors. Clinical scholars observe in practice environments to deliberate on feasible translations of best practices to their home country. These international exchange programs elevate the narrative of nursing by strengthening nursing’s professionalism and building synergies in research, education and practice, benefitting health systems globally.

## Conclusion

The WHO celebration of the Year of the Nurse and the Midwife boosts narrative change for these professions. Innovative approaches being practiced in academic nursing schools in the areas of global learning, interprofessional educational approaches, and partnership are also driving efforts to change the narrative on nursing while advancing the science of nursing through innovative research, practical learning, and commitment to health equity for both local and global communities. Given nurses’ numbers and critical role in the delivery of essential health services and in strengthening the health system worldwide, these efforts are essential to disrupt narratives that perpetuate negative perspectives and to replace them with counter-narratives that elevate, motivate and empower the professions.
